# Editorial: Sustainable solutions in food technology, volume II

**DOI:** 10.3389/fnut.2023.1150768

**Published:** 2023-02-06

**Authors:** Rui M. S. Cruz, Jooyeoun Jung

**Affiliations:** ^1^Department of Food Engineering, Institute of Engineering, Universidade do Algarve, Faro, Portugal; ^2^MED-Mediterranean Institute for Agriculture, Environment and Development and CHANGE-Global Change and Sustainability Institute, Faculty of Sciences and Technology, Universidade do Algarve, Faro, Portugal; ^3^Department of Food Science and Technology, Oregon State University, Corvallis, OR, United States

**Keywords:** food, sustainability, waste recovery, green technologies, quality

This is the second edition of the Research Topic—*Sustainable Solutions in Food Technology* and it consists of four articles that provide recent advances and insights on new technologies and food sources that ensure food quality and safety while also having a positive environmental impact.

The article of Moreira et al. presents pequi (*Caryocar brasiliense*) waste extract as a synergistic agent in the microbial and physicochemical preservation of low-sodium raw goat cheese. To investigate the effect of pequi waste extract (PWE), combined with UV-C radiation (CEU) and vacuum packaging (CEV) a Principal Component Analysis was applied. The results showed that CEV samples presented lower loadings for *Enterobacteriaceae* and *Staphylococcus* subsp. compared with other treatments. A count reduction up to 3-fold (*p* < 0.05) was observed compared to when vacuum treatment was used alone. On the other hand, CEU presented an increase of up to 1.2-fold in staphylococcal count compared to the treatment only with UV-C. A 8.5% protein loss was shown when PWE was added to UV-C-treated cheeses. During storage, PWE, particularly in CEV, delayed post-acidification. CEV was more stable for color and texture, up to 4.5 and 1.6-fold, respectively, compared with the vacuum treatment. The obtained results indicate that PWE, when combined with the vacuum, may be a new and promising synergistic agent in the microbial and physicochemical preservation of low-sodium raw milk cheese.

Albertos et al. showed the characterization of Zamorano-Leonese donkey milk. In terms of amino acid and protein profile the studied breed was similar from others. However, a higher content of unsaturated fatty acids (around 55%) was presented, and the main contributor was oleic fatty acid (25%). The milk also showed a higher content of vitamin C (63 mg/L), riboflavin (345 μL/L), folic acid (526 μL/L), and vitamin E (7 μg/100 g) compared with other donkey breeds. A high concentration of vitamin D (1.5 μg/100 g) was also observed, although with a low-fat content. On the other hand, this breed presented a lower mineral concentration and ash content. Regarding the micronutrients, high amounts of zinc (2,185 μg/kg) and selenium (107 μg/kg) were detected. This milk, besides being a good source of protein, is a food and/or ingredient that may contribute for a suitable functioning of the circulatory and immune systems.

Ma et al. presented the influence of multi-frequency ultrasound-assisted immersion freezing (UIF) on cultured large yellow croaker (*Larimichthys crocea*). The results showed that UIF-175-treated samples presented a total freezing time, lower 29.71% than the IF-treated samples (*p* < 0.05). The UIF contributed for a reduction in the thawing and cooking losses, and total volatile base nitrogen (TVB-N), and an increase in the water holding capacity (WHC). At 175 W the samples showed minimum thawing loss. The results were 53.11% lower compared with the AF (air freezing) treated samples and 46.45% lower than the IF (immersion freezing) treated samples (*p* < 0.05). The lowest cooking loss (10.28%) was presented by the UIF-175-treated samples, similar to the fresh samples (9.30%). The same treated samples also presented the highest WHC (81.33%) and again similar to the fresh ones (85.78%) (*p* > 0.05). In what concerns the TVB-N, UIF-175 samples showed similar values to fresh samples (10.10 mg N/100 g), and lower than the samples frozen by other ultrasonic treatments. The multi-frequency UIF at 175 W also reduced ice crystal size and pore diameter. A reduction in the accumulation of bitter amino acids and an increase in the accumulation of umami amino acids was also observed for the UIF-175 treatment. The quality of large yellow croaker can definitely be improved by multi-frequency UIF, regulated at a suitable ultrasonic power.

Cortes-Ferre et al. showed an enzyme-assisted extraction (EAE) of anti-inflammatory compounds from habanero chili pepper (*Capsicum chinense*) seeds (CPSs). The objective of this study was to define the cellulase-assisted extraction conditions of capsaicinoids and phenolic compounds from habanero CPSs and to evaluate the anti-inflammatory activity of the obtained extracts on murine macrophages. The conditions, 30 °C, 2,500 UI/L, and 150 min of extraction presented the highest phenolic compound content (337.96 mg GAE/L). At 45 °C with 250 UI/L for 150 min, the highest capsaicin content (310.23 μg/mL) was observed, while at 60 °C, 2,500 UI/L, and 120 min the results obtained for dihydrocapsaicin were 167.72 μg/mL. The conditions 60 °C, 250 UI/L, and 150 min presented the highest anti-inflammatory response, and nitric oxide production was reduced to 22.56%. EAE, and using water as a solvent, allowed the recovery of compounds with anti-inflammatory activity from habanero CPSs.

## Author contributions

RC and JJ contributed to manuscript writing, revision, and reading. All authors contributed to the article and approved the submitted version.

